# Regenerative peptides for inferior alveolar nerve injury: a narrative review

**DOI:** 10.3389/fdmed.2026.1977522

**Published:** 2026-10-27

**Authors:** Scott Froum, Nathan Estrin, Gianna Pico, Mikhail Yakubov, Paras Ahmad, James LaValle

**Affiliations:** 1 Department of Periodontics, School of Dental Medicine, Stony Brook University, Stony Brook, NY, United States; 2 Lake Erie College of Osteopathic Medicine, School of Dental Medicine, Bradenton, FL, United States; 3 Private Practice, NYC, NY, United States; 4 College of Dental Medicine, Lincoln Memorial University, Knoxville, TN, United States; 5 NewYork-Presbyterian Hospital, NYC, NY, United States; 6 American Academy of Anti-Aging Medicine (A4M), Boca Raton, FL, United States

**Keywords:** ARA-290, BPC-157, inferior alveolar nerve, peptides, peripheral nerve injury, peripheral nerve regeneration, TB-500

## Abstract

Inferior alveolar nerve (IAN) injury is a clinically important complication of oral and maxillofacial procedures that can lead to persistent neurosensory impairment, and effective regenerative interventions are presently scarce. This narrative review assessed the mechanistic rationale, preclinical evidence, translational limitations, and potential future relevance of regenerative peptides for IAN repair. Particular attention is given to BPC-157, thymosin *β*4 (T*β*4) and its related synthetic fragment TB-500, and ARA-290 (cibinetide), whose reported biological functions intersect with primary mechanisms implicated in peripheral nerve regeneration, such as cellular survival, Schwann cell activity, inflammatory modulation, angiogenesis, extracellular matrix remodeling, and axonal regeneration. Nevertheless, the available evidence is mainly sourced from pre-clinical and non-IAN systems, and direct evidence of efficacy in iatrogenic or traumatic IAN injury remains inadequate. BPC-157 and TB-500 remain investigational compounds with important safety, pharmacokinetic, and regulatory uncertainties, whereas evidence associated with TB-500 should be differentiated from that obtained with full-length T*β*4. Translation to IAN injury is further complicated by the lack of validated local delivery approaches, uncertain target-site pharmacokinetics, the anatomically restricted mandibular canal, and injury severity. Potential combination strategies involving traditional therapies, biomaterial-assisted delivery frameworks, and dental stem cell-mediated regenerative approaches are mechanistically plausible but remain hypothesis-generating rather than definitive synergistic interventions. Prospective research should prioritize IAN-relevant experimental systems, injury-severity classification, standardized outcome measures, target-site pharmacokinetics, and rigorously controlled clinical trials. Overall, regenerative peptides constitute a biologically plausible but currently investigational method for IAN repair, and their clinical application will rely on exhibiting reproducible efficacy and safety within indication-specific models and human trials.

## 1 Introduction

Inferior alveolar nerve (IAN) injury is an inherent risk in a range of surgical and dentoalveolar procedures, such as third-molar extraction, dental implant placement, endodontic over-instrumentation, and orthognathic surgery (1). While most of the injuries are mild and self-limiting, a clinically substantial subset leads to persistent neurosensory dysfunction, such as paresthesia, dysesthesia, or complete anesthesia of the lower lip and chin (2). These deficits can significantly impact speech, mastication, and psychosocial well-being (3). Although peripheral nerves retain an inherent regenerative capability, functional recovery relies on synchronized vascular, inflammatory, cellular, and structural responses; dysregulation of these mechanisms may lead to maladaptive or partial repair (4–6).

Presently, IAN injury management remains mainly reactive, focusing on symptom control, secondary inflammation mitigation, or delayed microsurgical therapy instead of active biological regeneration (7). Regenerative peptides provide a conceptually unique strategy since their impacts may extend across intertwined mechanisms implicated in peripheral nerve repair, such as angiogenesis, immune modulation, Schwann cell (SC) responses, extracellular matrix (ECM) remodeling, mitochondrial homeostasis, and axonal regeneration (8). This multimodal biological activity offers the rationale for assessing peptide-mediated approaches in craniofacial nerve injury.

Notwithstanding growing interest in peptide therapeutics, the existing literature remains fragmented across basic science, experimental animal models, and isolated clinical studies, with limited focus on their significance to IAN injury. Additionally, the unique biological and anatomical features of the mandibular canal, such as restricted vascular reserve, limited osseous structure, and risk of fibrosis, create regenerative difficulties that vary significantly from those found in other peripheral nerve models (9–11). Hence, a synthesis linking IAN injury pathophysiology with emerging peptide biology is required.

This review critically assesses the mechanistic basis, preclinical evidence, translational limitations, and potential clinical relevance of regenerative peptides for IAN injury. Beyond summarizing the existing evidence, we propose a biologically integrated system that corroborates candidate peptide-based therapies with the temporal phases of peripheral nerve repair, hence offering a conceptual basis for future translational research in oral and maxillofacial surgery.

## 2 Pathophysiology of inferior alveolar nerve injury

IAN injury propagates a temporally synchronized event of degenerative and regenerative mechanisms that is strongly affected by the anatomical limitations of the mandibular canal (12, 13). Inside the restricted osseous environment, edema, ischemia, and fibrosis may augment the outcomes of the primary insult. Chemical injury from endodontic materials, thermal injury during osteotomy or mechanical trauma from implant placement or drilling can disturb axonal integrity, injure SCs, and undermine the surrounding neurovascular microenvironment (14). The resultant regenerative response involves Wallerian degeneration, immune cell recruitment, SC rewiring, revascularization, and axonal regrowth (Figure 1) (12, 15).

**Figure 1:**
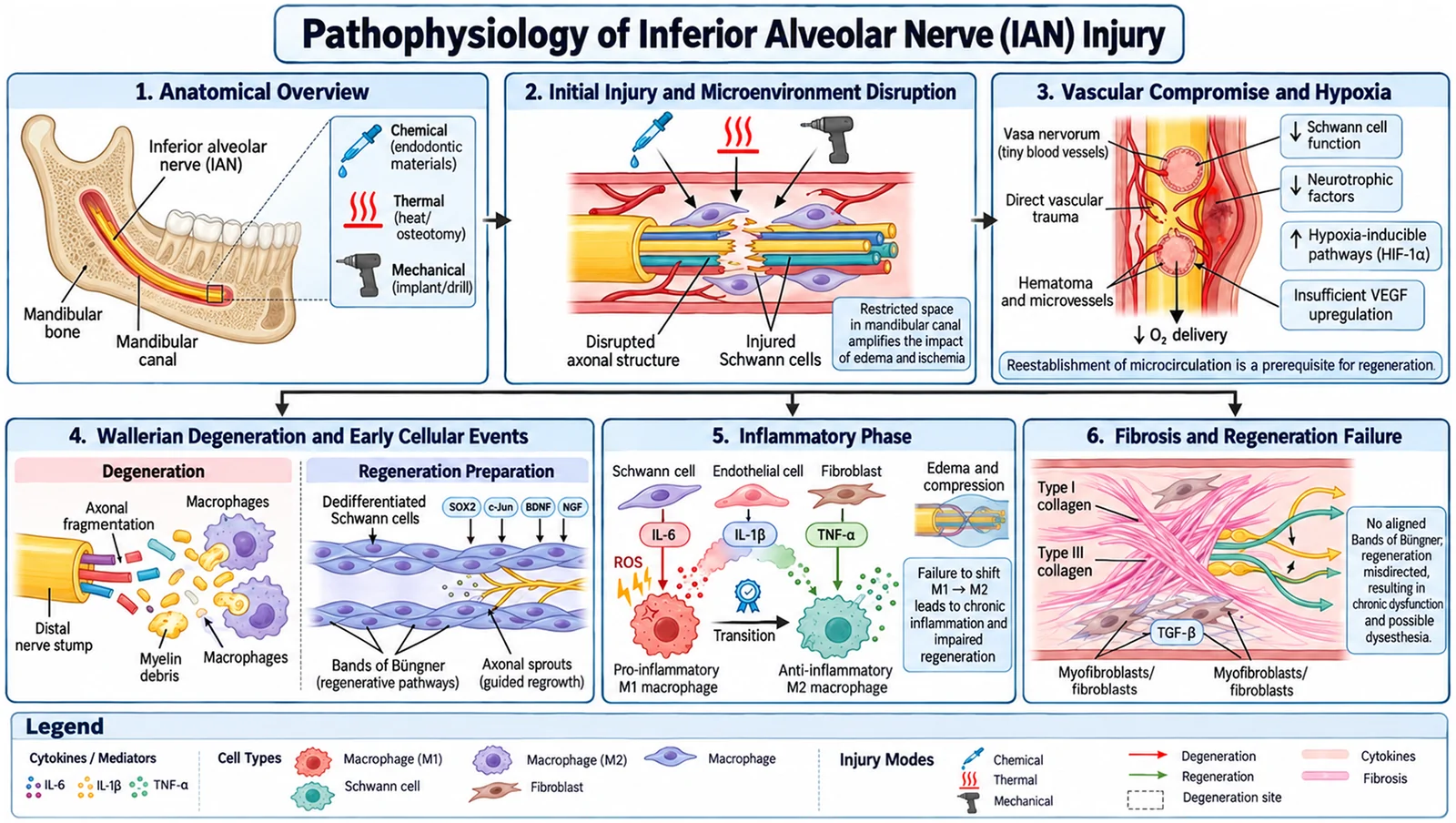
Schematic overview of the pathophysiology of IAN injury. After chemical, thermal, or mechanical insult, IAN injury propagates axonal disruption, Schwann cell damage, vascular compromise, Wallerian degeneration, and inflammatory responses. Persistent hypoxia and dysfunctional immune signaling enhance fibrosis, compromise axonal guidance, and eventually undermine functional nerve regeneration.

### 2.1 Classification and severity of IAN injury

The magnitude of biological response and the possibility of spontaneous recovery following an IAN injury rely markedly on the severity and structural degree of neural damage. Peripheral nerve injuries are generally classified utilizing the Seddon and Sunderland classifications, which offer additional ways for associating structural disruption with regenerative potential. Seddon widely divides injuries into neurotmesis, axonotmesis, or neurapraxia, while Sunderland further classifies injury into five degrees based on the gradual disruption of the axon and surrounding epineurial, perineural, and endoneural structures (16, 17).

Sunderland grade I relates to neurapraxia, wherein axonal continuity remains intact, and the primary disruption is a transient conduction block linked with focal demyelination; spontaneous recovery is usually anticipated (18). Grade II constitutes axonal disruption with maintenance of the endoneurial tubes and follows distal Wallerian degeneration, but the preserved connective tissue structure offers a way for axonal regrowth (18). Grades III and IV correspond to gradually greater abnormality of the endoneurium and perineurium, respectively, leading to increased intraneural dysregulation, fibrosis, misdirected axonal regeneration, and a gradually less predictable capability for spontaneous functional recovery (18). Grade V represents neurotmesis, with complete loss of nerve continuity and poor spontaneous recovery, typically requiring surgical repair (Table 1) (18).

**Table 1:** Comparative mechanisms, potential therapeutic roles, advantages, limitations, and evidence status of conventional pharmacological agents and investigational peptides for IAN injury.

Treatment	Primary mechanism(s)	Potential role in IAN injury	Advantages	Disadvantages/limitations	Evidence status
Corticosteroids (dexamethasone)	Glucocorticoid receptor-based inhibition of pro-inflammatory cytokines & COX/prostaglandin signaling; ↓ vascular permeability & perineural edema	Early limitation of inflammation, edema, compression & secondary neural injury	Clinically familiar; rapid anti-inflammatory action; may preserve the neural microenvironment when used early	Does not directly enhance nerve regeneration; extended/excessive inhibition may interfere with macrophage-based debris clearance, SC activity, angiogenesis & ECM remodeling; benefit is time-mediated	Established clinical use for inflammatory control, but regenerative effect is limited
Local anesthetics (bupivacaine)	Voltage-gated Na⁺ channel blockade; inhibition of peripheral nociceptive transmission & potentially neurogenic inflammation/central sensitization	Perioperative pain control & modulation of nociceptive signaling	Established clinical use; effective analgesia; may limit peripheral & central sensitization	Limited direct effect on structural nerve regeneration; does not directly enhance axonal growth, SC function, or angiogenesis	Established for analgesia; regenerative role remains limited
BPC-157	VEGF/eNOS & PI3 K/AKT-associated signaling; NO regulation; modulation of inflammatory & ECM responses	Potential support of microvascular restoration, cytoprotection, inflammatory regulation & organized tissue repair	Multimodal activity; potentially addresses vascular, inflammatory & fibrotic components simultaneously	Limited IAN-specific evidence; optimal dose, delivery, pharmacokinetics, long-term safety & regulatory status remain unresolved	Predominantly preclinical
Thymosin β4/TB-500	Actin/cytoskeletal regulation; promotion of cellular migration; angiogenic & ECM-modulating effects	Potential enhancement of SC migration, axonal elongation, vascular repair & limitation of fibrosis	Direct relevance to cellular organization & structural repair; combines cytoskeletal, vascular & antifibrotic actions	Limited craniofacial/IAN-specific evidence; dosing, delivery, long-term safety & translation to humans remain uncertain	Predominantly preclinical
ARA-290 (cibinetide)	Activation of the innate repair receptor; immunomodulation; inhibition of inflammatory/oxidative pathways; mitochondrial cytoprotection	Potential control of maladaptive neuroinflammation while supporting tissue repair & neural survival	More targeted immunomodulatory approach; avoids erythropoietic effects of EPO; has some human neuropathy data	Evidence largely derives from non-IAN neuropathies; optimal IAN-specific dose, route, timing & efficacy are unknown	Preclinical evidence plus limited human evidence in other neuropathic conditions

AKT, protein kinase B; COX, cyclooxygenase; ECM, extracellular matrix; eNOS, endothelial nitric oxide synthase; EPO, erythropoietin; IAN, inferior alveolar nerve; IL, interleukin; IRR, innate repair receptor; NO, nitric oxide; PI3 K, phosphoinositide 3-kinase; SC, Schwann cell; Tβ4, thymosin β4; TB-500, thymosin β4-derived peptide; TNF-α, tumor necrosis factor-alpha; VEGF, vascular endothelial growth factor.

This differentiation is especially significant for regenerative methods discussed in this study. Biological regulation of microvascular perfusion, inflammation, SC functionality, or ECM remodeling may theoretically be most relevant when adequate neural architecture remains to facilitate endogenous regeneration (19, 20). Contrarily, in high-grade injuries representing complete nerve discontinuity or severe fascicular disruption, peptide-based biological support would not be anticipated to substitute for microsurgical reconstruction. Therefore, injury grade should be regarded as a crucial stratification variable in prospective research assessing regenerative peptides for IAN injury.

### 2.2 Wallerian degeneration and initial cellular response

In cases of injuries involving axonal disruption (i.e., Sunderland grades II-V), the distal axonal segment undergoes Wallerian degeneration, a highly coordinated mechanism that prepares the microenvironment for regeneration (21). Within hours to days, axonal cytoskeleton elements, such as microtubules and neurofilaments, disintegrate, resulting in fragmentation of the axon (22). Simultaneously, myelin sheaths break down into lipid-rich debris that must be effectively cleared to allow axonal regrowth (22). SCs play a pivotal role in this mechanism (23). Upon injury, they undergo rapid dedifferentiation from a myelinating phenotype into a “repair” phenotype characterized by: (i) downregulation of myelin-related genes (MBP, MPZ); (ii) upregulation of regeneration-related genes (SOX2, c-Jun); and (iii) release of neurotrophic factors, including brain-derived neurotrophic factor (BDNF) and nerve growth factor (NGF) (24, 25). These repair SCs proliferate and align longitudinally to produce Bands of Büngner, which function as guiding structures for regenerating axonal sprouts (26). Concurrently, circulating macrophages infiltrate the injury region and collaborate with SCs to phagocytose myelin and axonal debris (27). Crucially, the effectiveness of Wallerian degeneration is a central determinant of regenerative success (27). Partial or delayed clearance of inhibitory myelin-related proteins can undermine axonal extension (28). Furthermore, the structural maintenance of the endoneurial tubes must be preserved; disruption of this architecture can result in misdirected axonal growth and aberrant sensory recovery (Figure 1) (28).

### 2.3 Inflammatory pathways and secondary injury

The inflammatory response is inevitable for the propagation of repair, but must be tightly modulated to avert further tissue injury. After IAN injury, local cells, including SCs, endothelial cells (ECs), and fibroblasts, secrete pro-inflammatory cytokines, including interleukin (IL)-6, IL-1β, and tumor necrosis factor–*α* (TNF-α) (29–31). These regulators improve immune cell recruitment and stimulation but also contribute to a pro-oxidative microenvironment (32). Macrophages, which initially adopt a pro-inflammatory (M1) phenotype, are important for debris clearance (33). Nevertheless, extended M1 predominance may lead to: (i) prolonged cytokine secretion; (ii) increased generation of reactive oxygen species (ROS); and (iii) mitochondrial dysregulation and lipid peroxidation (34, 35). Shift to an anti-inflammatory (M2) phenotype is imperative for the resolution of inflammation and advancement to tissue healing (36). Failure of this phenotypic transition has been correlated with chronic inflammation and compromised regeneration (37). Moreover, excessive inflammation hinders the nerve-blood barrier, increasing vascular permeability and resulting in perineural edema (38). In the limited space of the mandibular canal, even modest swelling can lead to substantial compression of the nerve, further undermining axonal transport and conduction (39). This process contributes to secondary neural injury, whereby damage extends beyond the initial insult via inflammatory and ischemic pathways (Figure 1) (40).

### 2.4 Vascular compromise and hypoxia

Sufficient vascular supply is a basic need for nerve regeneration (41). The IAN is supplied by a delicate network of microvessels, the vasa nervorum, that provide oxygen and metabolic substrates required for axonal growth and SC activity (41). Injury-provoked vascular disruption transpires via several pathways: (i) direct mechanical injury to microvessels; (ii) endothelial dysregulation regulated by inflammatory cytokines; and (iii) compression from hematoma or edema (42). These modifications decrease perfusion and generate a hypoxic niche. Hypoxia has numerous deleterious effects on nerve repair, such as: (i) undermined SC proliferation and differentiation; (ii) decreased production of neurotrophic factors; and (iii) stimulation of hypoxia-inducible pathways that may favor fibrosis over regeneration (41, 43). Simultaneously, hypoxia can induce compensatory angiogenic pathways, especially via the upregulation of vascular endothelial growth factor (VEGF) (44). Nevertheless, this response is mostly inadequate or dysregulated in the setting of ongoing inflammation and tissue injury (44). The association between neural damage and vascular insufficiency is increasingly recognized as a pivotal determinant of outcome. Indeed, reestablishment of microcirculation is not only supportive but may be a prerequisite for successful axonal regeneration (Figure 1) (44). These vascular and anatomical features also have direct consequences for therapeutic delivery. The restricted intrabony environment may confine the volume that can safely be introduced locally, while injury-associated modifications in microvascular perfusion may affect both systemic delivery to the nerve and clearance of locally delivered agents.

### 2.5 Fibrosis and regenerative failure

One of the primary hurdles to effective nerve regeneration is the fibrotic scar tissue formation within and around the injured nerve (45). Fibrosis originates from the stimulation of myofibroblasts and fibroblasts, mediated by transforming growth factor-β (TGF-β) and other profibrotic regulators (46). Excess ECM formation, especially collagen types I and III, results in: (i) mechanical disruption of axonal extension; (ii) dysregulation of endoneurial architecture; and (iii) modified biochemical pathways within the regenerative microenvironment (47). Regarding IAN damage, fibrosis is especially problematic owing to the limited anatomy of the mandibular canal (1). Even minimal scar formation can increase intraneural pressure, compress regenerating axons, and compromise reinnervation of target tissues (45). Moreover, fibrotic tissue is devoid of the aligned, permissive architecture of Bands of Büngner, resulting in misdirected or disorganized axonal growth. Clinically, this may manifest as partial sensory recovery or dysesthetic sensations (Figure 1) (45).

## 3 Current and traditional management of IAN injury

Contemporary management of IAN injury is directed mainly toward restricting secondary neural damage, regulating inflammation and pain, and maintaining conditions conducive to spontaneous recovery or resultant treatment (19). Among the pharmacological methods utilized during the early post-injury period, corticosteroids and local anesthetics primarily target nociceptive and inflammatory cascades instead of directly activating structural nerve regeneration (48). Comprehending the processes, treatment advantages, and drawbacks of these traditional strategies offers a vital clinical context for assessing emerging regenerative approaches (49). Notably, management and prognosis are also based on the structural severity of injury, i.e., lower-grade injuries with preserved neural structure may recover spontaneously, while high-grade Sunderland IV-V injuries may mandate surgical exploration and reconstruction instead of pharmacological management alone (9).

### 3.1 Corticosteroids: timing matters

Corticosteroids, especially dexamethasone, remain a cornerstone of acute management after suspected nerve injury in oral and dental surgical procedures (50). Their main therapeutic importance lies in their capability to mitigate the initial inflammatory pathways that contribute to secondary neural injury (51). Mechanistically, corticosteroids exert their effects via glucocorticoid receptor-mediated transcriptional mediation, leading to: (i) downregulation of pro-inflammatory cytokines (IL-6, IL-1β, TNF-α); (ii) suppression of cyclooxygenase (COX) signaling and prostaglandin production; and (iii) stabilization of lysosomal membranes and decrease in cellular edema (52). By alleviating perineural edema and vascular permeability, corticosteroids may mitigate mechanical compression, restore microcirculatory flow, and preserve axonal conduction in the early post-injury period (51). Moreover, corticosteroids may regulate the nerve-blood barrier, restrict extravasation of inflammatory regulators and further protect the neural niche (53). Clinically, this translates into decreased pain, improved neurosensory activity, and potentially alleviated risk of advancement to chronic neuropathy when applied promptly (54). Nevertheless, the advantages of corticosteroids are highly time-mediated (55). Their application seems greatest during the acute inflammatory stage, usually within hours to days after injury. Excessive or extended corticosteroid exposure may have unintended manifestations, such as: (i) inhibition of macrophage functionality required for debris clearance; (ii) suppression of SC proliferation and differentiation; and (iii) undermining of angiogenesis and ECM remodeling (55–57). These influences may eventually obstruct the shift from inflammation to regeneration (58). Therefore, corticosteroids should be conceptualized not as regenerative agents, but as early-stage regulators of secondary injury, with a therapeutic window that necessitates prudent clinical assessment (57). These temporal constraints highlight the difference between early regulation of secondary injury and active enhancement of resultant regenerative mechanisms (Figure 2) (59).

**Figure 2:**
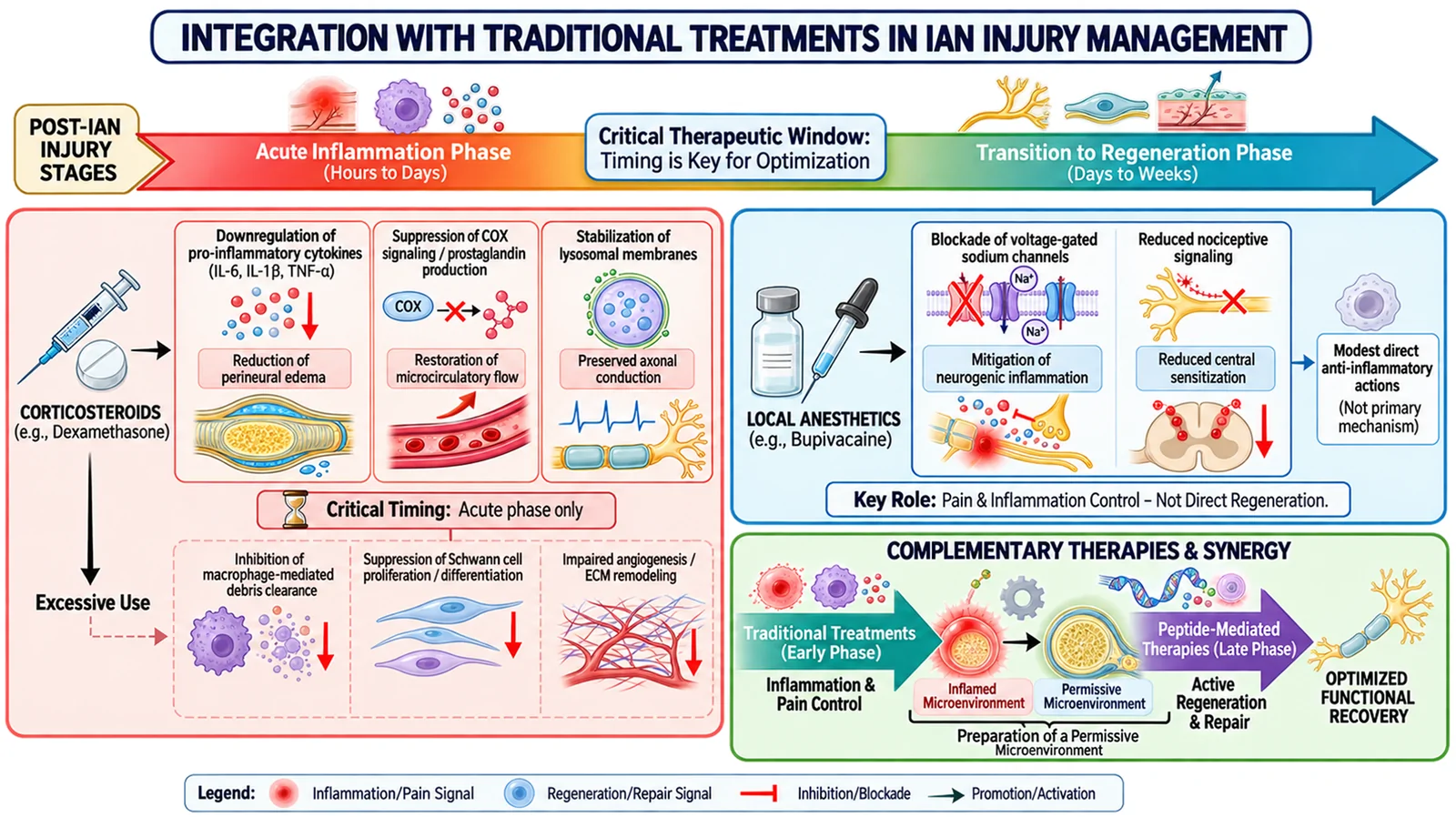
Mechanistic overview of peptide-mediated regenerative interventions for IAN injury. Compared with classical single-target treatments, BPC-157, thymosin β4 (TB-500), and ARA-290 enhance nerve repair via complementary impacts on angiogenesis, immune regulation, Schwann cell activity, ECM remodeling, and cytoprotection, overall generating a regenerative niche that facilitates functional recovery.

### 3.2 Local anesthetics: beyond analgesia

Long-acting local anesthetics, including bupivacaine, are regularly utilized in dental practice for perioperative pain control. Nevertheless, emerging evidence indicates that their contribution to nerve injury management may extend beyond analgesia, affecting both central and peripheral elements of the pain response (60). At the peripheral level, local anesthetics block voltage-gated sodium channels, hence suppressing the action potential initiation along nociceptive fibers. This hindrance of nociceptive signaling may have numerous downstream impacts: (i) a decrease in neurogenic inflammation regulated by substance P and calcitonin gene-related peptide (CGRP); (ii) reduction of peripheral sensitization of damaged nerve fibers; and (iii) mitigation of inflammatory feedback loops within the local tissue niche (61). By restricting nociceptive input from the injury site, local anesthetics may indirectly decrease the secretion of pro-inflammatory regulators, hence contributing to a more controlled inflammatory response (62). At the central level, prolonged nociceptive input after nerve injury can result in central sensitization, characterized by increased excitability of dorsal horn neurons and augmentation of pain signaling (63). This mechanism is the central contributor to the formation of chronic neuropathic pain. Extended neural inhibition with agents, including bupivacaine, may help hinder this process, mitigating the probability of long-term pain syndromes (63). Moreover, some investigations indicate that local anesthetics may exert direct anti-inflammatory impacts, such as the regulation of immune cell functionality and cytokine release. Nonetheless, these influences are comparatively modest and not fully characterized in the context of nerve regeneration (64). It is noteworthy that although local anesthetics may contribute to enhanced patient comfort and regulation of pain signaling, their role in structural nerve repair remains restricted. Hence, even though local anesthetics offer crucial regulation of pain and nociceptive pathways, their contribution to structural nerve repair seems restricted, as they do not directly enhance central regenerative cascades, including SC activity, axonal growth, or angiogenesis (Figure 2) (64).

## 4 Mechanistic rationale for peptide-based regenerative therapies

The drawbacks of traditional strategies in directly enhancing structural nerve repair offer a rationale for assessing biologically regenerative approaches. The pathophysiology of IAN injury presents many potential therapeutic targets transcending acute inflammation and pain, such as cytoprotection, SC activity, revascularization, ECM remodeling, and axonal regeneration. Candidate regenerative peptides may impact numerous of these pathways concurrently (65–67). The following sections analyze BPC-157, thymosin *β*4/TB-500, and ARA-290 based on their primary mechanistic actions and potential relevance to IAN repair (Figure 3).

**Figure 3:**
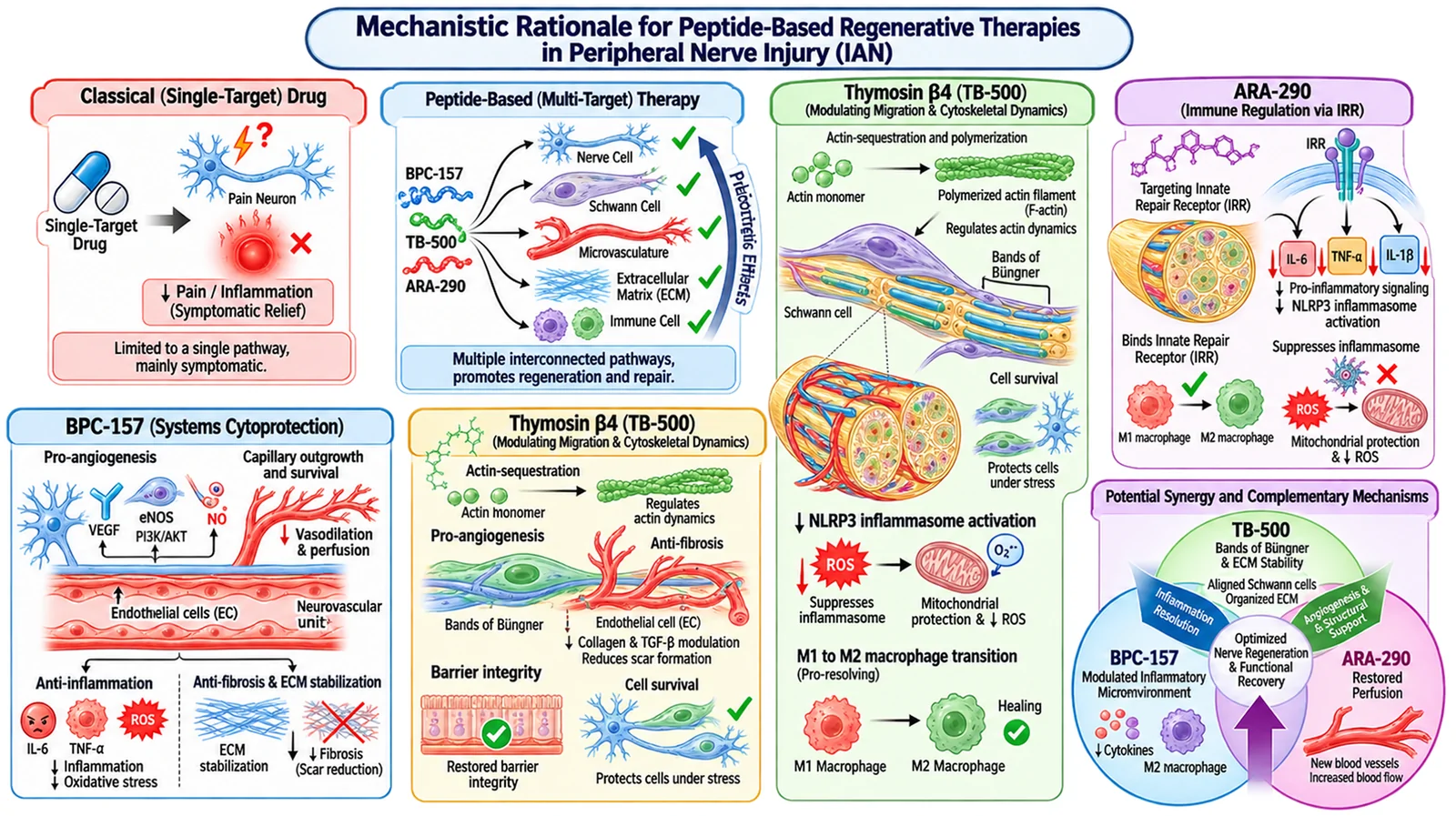
Integration of traditional and regenerative interventions for IAN injury. Corticosteroids and local anesthetics mainly alleviate acute inflammation, edema, and pain during the early post-injury stage, whereas subsequent peptide-based treatments aim to promote regeneration by generating a permissive niche for nerve repair and functional recovery.

### 4.1 BPC-157: preclinical rationale and limitations for neural repair

BPC-157 (Body Protection Compound-157) is an investigational synthetic pentadecapeptide sourced from a gastric protein sequence that has been investigated mainly in preclinical systems of vascular, gastrointestinal, musculoskeletal, and other forms of tissue injury (68, 69). Its inclusion in this review is not dependent on established clinical potential in neural regeneration, but on the intersection of its reported neurovascular activities with a restricted body of direct experimental evidence in peripheral nerve injury. In a rodent sciatic nerve transection model, BPC-157 delivery was related to enhanced histomorphometric parameters of axonal regeneration and myelination, elevated vascular representation, enhanced electrophysiological healing, and better functional recovery (70). Nonetheless, these findings arise from preclinical models, have not been defined in the IAN, and mandate independent replication and validation before they can be assessed in IAN injury.

Mechanistically, BPC-157 has been related to the regulation of VEGF-associated angiogenic pathways and endothelial nitric oxide synthase (eNOS)/NO interplay, with reported impacts on endothelial survival and microvascular reestablishment (68, 71, 72). These vascular impacts offer an indirect rationale for assessing BPC-157 in IAN injury since dysregulation of the vasa nervorum, ischemia, and compromised microcirculation are pivotal elements of secondary neural injury within the mandibular canal. Additionally, BPC-157 has been related to experimental models with regulation of inflammatory pathways, fibroblast functionality, oxidative stress, and ECM remodeling (Figure 3) (68, 73).

Notably, BPC-157 is not approved by the U.S. Food and Drug Administration (FDA) for human therapeutic application, and its clinical safety, effective dose, pharmacokinetics, and long-term toxicity remain inadequately defined (74). FDA has detected potential safety issues related to compounded BPC-157, such as immunogenicity and uncertainties associated with peptide impurities and active pharmaceutical ingredient characterization (74). Moreover, BPC-157 is classified by the World Anti-Doping Agency (WADA) under the S0 category of non-approved substances, and it is prohibited in sport (68). Hence, its discussion here should be considered only within an investigational and hypothesis-generating system. Presently, the evidence does not support clinical use of BPC-157 for IAN injury, and its significance in this context lies mainly in detecting tissue-healing and neurovascular signaling that may mandate future experimental research.

### 4.2 Thymosin β4 (TB-500): neuroregenerative rationale, molecular distinction, and safety considerations

Thymosin β4 (Tβ4) is an endogenous actin-binding peptide composed of 43 amino acids, with biological activities extending beyond angiogenesis, such as the modulation of cell migration, cytoskeletal organization, inflammation, tissue remodeling, and neural repair (75). Notably, Tβ4 should be differentiated from TB-500, a synthetic Tβ4-associated fragment that is mostly discussed together with the parent peptide but for which the direct experimental and clinical evidence base is considerably more scarce (76). Most of the regenerative literature usually extrapolated to TB-500 has, in fact, assessed full-length T*β*4 rather than the TB-500 fragment (77). Hence, the biological rationale considered in the present study originates primarily from reports of Tβ4 and should not be considered to exhibit equivalent efficacy or activity to TB-500.

The justification for considering Tβ4 in neural repair transcends its angiogenic features. In experimental diabetic peripheral neuropathy, Tβ4 therapy enhanced functional vascular density and local blood flow within the sciatic nerve and was linked to enhanced neurological activity, with mechanistic impacts involving both ECs and SCs via the PI3 K/AKT- and Ang1/Tie2-related pathways (78). Moreover, in experimental diabetic neuropathy, extended Tβ4 therapy has been related to enhanced sensory and motor nerve conduction, elevated intraepidermal nerve fiber density, maintenance of myelin and axonal organization, and improved neurite outgrowth (79). Other experimental neurological systems have associated Tβ4 with oligodendroglial differentiation, cytoskeletal remodeling, and axonal regeneration (80). These outcomes offer a neurobiological justification for examining Tβ4-associated signaling in peripheral nerve repair; nonetheless, they arise mainly from non-IAN preclinical models and cannot currently establish efficacy in traumatic IAN injury.

Tβ4-dependent angiogenesis also mandates special caution (81). Even though the reestablishment of the vasa nervorum could theoretically support regeneration of an ischemic injured nerve, Tβ4 expression and signaling have also been related experimentally to tumor cell migration, tumor angiogenesis, and metastatic behavior (80, 82). Therefore, pro-angiogenic functionality cannot be assumed to be uniformly advantageous, especially when long-term safety, tissue distribution, exposure duration, and dose have not been established. Notably, these oncological findings predominantly concern Tβ4 expression or experimental manipulation and should not be considered evidence that therapeutic delivery of TB-500 has been shown to cause tumors in humans. Instead, they detect a biologically plausible safety issue that necessitates dedicated assessment before regenerative application.

For TB-500 particularly, the clinical evidence is significantly weaker. FDA (83) has found no human exposure data for drug products containing the thymosin β4 fragment (LKKTETQ), also called TB-500, and has underscored unresolved issues concerning safety, peptide-associated impurities, and immunogenicity. Additionally, Tβ4 and its derivatives, such as TB-500, are prohibited by WADA (84). Hence, TB-500 should currently be regarded as an investigational Tβ4-associated compound instead of a definitive neuroregenerative intervention. Its inclusion in the present study is aimed at differentiating it from the better-characterized parent peptide and to underscore both the potential significance and considerable uncertainty of Tβ4-associated signaling to IAN regeneration.

### 4.3 ARA-290: immune regulation via the innate repair receptor

ARA-290 (cibinetide) represents a mechanistically unique strategy within the spectrum of regenerative peptides, targeting the innate repair receptor (IRR), a heteromeric receptor complex stimulated during tissue damage (85). Sourced from erythropoietin (EPO), ARA-290 selectively retains anti-inflammatory and tissue-protective features without activating erythropoiesis, hence avoiding the hematologic risks related to EPO treatment (85). The stimulation of IRR propagates a cascade of intracellular signaling that collectively enhances cytoprotection and repair. These encompass: (i) inhibition of pro-inflammatory cytokine generation; (ii) suppression of NLRP3 inflammasome stimulation in SCs and macrophages; (iii) decrease in oxidative stress and mitochondrial dysregulation; and (iv) promotion of cell survival signaling (86). Unlike nonsteroidal anti-inflammatory drugs and corticosteroids, which extensively inhibit immune functionality, ARA-290 seems to recalibrate the inflammatory reaction, enhancing a transition from M1 to M2 phenotype (87). This differentiation is pivotal, as excessive immunosuppression can undermine debris clearance and delay regeneration (53). In preclinical and early clinical studies, ARA-290 has shown the capability to enhance small nerve fiber density and decrease neuropathic pain, indicating both functional and structural advantages (88, 89). Its impacts on mitochondrial preservation are especially important, since mitochondrial dysregulation is increasingly recognized as the central contributor to chronic neuropathy. Hence, ARA-290 can be considered an immune-centric regenerative regulator, targeting the inflammatory dysregulation that underlies secondary neural injury (Figure 3) (90).

## 5 Delivery approaches and pharmacokinetic hurdles within the mandibular canal

Translating regenerative peptides to IAN injury will rely not only on biological functionality, but also on whether therapeutically relevant concentrations can be reached and sustained within the injured nerve. This concern is especially crucial for the IAN since, unlike superficially accessible peripheral nerves, a considerable portion of the nerve lies within the mandibular canal (91). The rigid bony boundaries, restricted capability for tissue expansion, comparatively limited local vascular niche, and proximity to surgical and dental regions define a unique pharmacological environment (92). Hence, systemic exposure does not primarily predict peptide concentrations within the injured intrabony nerve, and traditional pharmacokinetic outcomes derived from other peripheral tissues may not be directly transferable to the IAN.

Systemic delivery is the least anatomically invasive approach and may be important for peptides whose biological functionality can be attained after systemic exposure (93). Nonetheless, this method has pivotal theoretical challenges for localized IAN injury, such as uncertainty pertaining to infiltration into the injured nerve and its microvasculature, off-target exposure, short peptide half-life and enzymatic breakdown, and dilution within the bloodstream (94). These concerns may require higher or repeated systemic dosing to sustain an effective local concentration, which could increase systemic exposure without guaranteeing adequate drug levels within the mandibular canal (95). Notably, the local pharmacokinetics of BPC-157, Tβ4/TB-500, and ARA-290 within the IAN or mandibular bone have not been defined.

Local delivery could enhance drug exposure at the injury location while restricting systemic exposure. In a dental clinic, the practicality of this method would rely heavily on injury mechanism and site. For surgically accessible injuries, including those identified during surgical exploration of an injured IAN, dental implant removal, or third molar surgery, a peptide-containing injectable hydrogel or other biodegradable depot could be positioned next to the affected nerve (14). Injectable hydrogels are theoretically attractive since they can conform to an irregular surgical defect and offer extended peptide release instead of the brief exposure associated with a simple solution injection (96). However, direct injection into the mandibular canal without surgical visualization would create considerable safety issues, such as injury to the accompanying inferior alveolar vessels, elevated intracanal pressure, hematoma formation, intraneural injection, and additional mechanical nerve injury (97). Hence, anatomically targeted or image-guided local delivery would necessitate rigorous preclinical validation before any clinical consideration.

Another local method constitutes the addition of regenerative peptides into biodegradable nerve membranes, conduits, or other extended-release biomaterials (98). Such platforms may be especially important when surgical exploration is already indicated for severe nerve discontinuity or when microsurgical repair or grafting is conducted. In this setting, the biomaterial could theoretically offer both controlled local release of a regenerative agent and structural guidance for regenerating axons. Nevertheless, the mandibular canal creates considerable dimensional limitations. Any depot or conduit must avoid excessive material volume, swelling, or inflammation mediated by degradation, since even modest increases in intracanal volume could worsen nerve compression and undermine the vasa nervorum (98). Therefore, delivery platforms introduced for peripheral nerves in compliant soft tissues cannot be assumed to be appropriate for the IAN without modification.

Local pharmacokinetics remain another unresolved concern. An ideal delivery platform would keep the peptide within a therapeutic concentration range throughout the relevant biological stage of nerve repair while averting an excessive early burst, extended exposure beyond the intended regenerative window, or rapid clearance from the surgical site (95). Injury-related changes in vascular permeability, biomaterial degradation kinetics, local vascular clearance, binding to ECM constituents, diffusion through the perineural environment, proteolytic breakdown, and peptide stability may all affect exposure at the IAN (93, 99). Presently, these parameters have not been properly defined for the candidate peptides in the bone-enclosed neural microenvironment (Table 2).

**Table 2:** Potential delivery approaches for regenerative peptides in IAN injury.

Delivery approach	Potential clinical setting	Potential advantage	Major limitation in IAN injury
Systemic administration	Injuries managed non-surgically	Non-invasive; no direct manipulation of injured nerve	Systemic dilution, degradation, off-target exposure, uncertain penetration into mandibular canal
Local solution/injection	Accessible injury site	High initial local concentration	Short residence time; risk of intraneural injection, hematoma, vascular injury & pressure
Injectable hydrogel/depot	Surgically accessible injury	Localized & potentially sustained release	Limited canal volume; swelling/degradation; uncertain release kinetics
Peptide-loaded conduit/scaffold	Severe injury undergoing microsurgical repair	Structural guidance plus controlled local delivery	Requires surgical access; geometric constraints; compression/foreign-body response
Biodegradable membrane/matrix	Exposed nerve during surgery	Localized delivery with reduced systemic exposure	Retention, degradation, dose control & tissue compatibility remain undefined

## 6 Mechanistic convergence and hypothetical combination approaches

While BPC-157, TB-500, and ARA-290 operate via unique primary pathways, their impacts converge on numerous vital axes of nerve repair (Figure 3):

*Inflammation regulation*: (a) ARA-290—targeted immunomodulatory signaling through IRRl and (b) BPC-157—mitigation of pro-inflammatory cytokines and oxidative stress (100).
*Angiogenesis and microvascular restoration*: (a) BPC-157—VEGF-regulated endothelial stimulation and NO signaling; and (b) TB-500—endothelial migration and capillary formation (72).
*Cell migration and structural organization*: (a) TB-500—actin-regulated SC and axonal movement; and (b) BPC-157—facilitates ECM stability (82).

Even though these pathways converge, this convergence should not be taken as evidence that combining regenerative peptides will yield synergistic or additive therapeutic impacts. Till today, controlled experimental reports assessing combinations of BPC-157, Tβ4/TB-500, and ARA-290 in IAN injury are scarce, and their pharmacokinetic and pharmacodynamic associations have not been defined. Theoretically, simultaneous delivery could yield additive, antagonistic, or unpredictable biological impacts, such as excessive regulation of inflammatory signaling, modified tissue remodeling, or unwanted associations with traditional pharmacological agents. Especial caution is required when combining agents with pro-angiogenic functionality. Both BPC-157 and Tβ4/TB-500 have been linked with angiogenic pathways (81, 82); hence, excessive or concurrent activation of these cascades could generate excessive or dysregulated neovascularization instead of regulated reestablishment of the microvasculature. In IAN injury, safety, durability, spatial organization, and dose-dependent interactions of such angiogenic impacts remain uninvestigated. Therefore, combinatorial interventions should be considered solely hypothesis-generating concepts that mandate dedicated safety, dose-ranging, and association investigations before clinical implementation (Figure 3).

Conventional and peptide-mediated treatments vary not only in their molecular targets but also in their proposed therapeutic roles and level of clinical evidence (101). Traditional methods mainly address acute inflammation and nociceptive pathways (102), while the regenerative peptides discussed in this review have been assessed for their potential to govern structural, inflammatory, cellular, and vascular elements of nerve repair (70). Nevertheless, these mechanistic differences should not be interpreted as evidence of superior clinical efficacy. Direct comparative trials in IAN injury are scarce, and associations between regenerative peptides and traditional agents have not been assessed. Hence, any proposed integration of these strategies remains conceptual instead of evidence-based. Their primary features, potential benefits, and limitations are presented in Table 3.

**Table 3:** Seddon and Sunderland classifications of peripheral nerve injury and their relevance to IAN regeneration.

Seddon classification	Sunderland grade	Structural injury	Wallerian degeneration	Expected spontaneous recovery	Relevance to regenerative approaches
Neurapraxia	I	Transient conduction block/focal demyelination; axon & connective-tissue architecture remain intact	No	Generally favorable/complete	Endogenous recovery usually predominates; supportive modulation of the local inflammatory/neurovascular environment may be sufficient
Axonotmesis	II	Axon disrupted; endoneurium, perineurium & epineurium preserved	Yes	Generally favorable, although recovery requires axonal regrowth	Preserved endoneurial pathways provide structural guidance for regeneration; biological support could theoretically facilitate the regenerative microenvironment
Axonotmesis	III	Axon & endoneurium disrupted; perineurium preserved	Yes	Variable; incomplete or misdirected recovery may occur	Loss of endoneurial guidance increases risk of disorganized regeneration and fibrosis
Axonotmesis	IV	Axon, endoneurium & perineurium disrupted; epineurium remains intact	Yes	Poor without intervention	Severe internal disorganization/neuroma-in-continuity; regenerative agents alone are unlikely to restore architecture
Neurotmesis	V	Complete disruption of the nerve & supporting connective-tissue layers	Yes	Poor to absent without surgical repair	Structural continuity must generally be restored surgically; peptide-based therapy, if eventually validated, could only be considered adjunctive

## 7 Dental stem cells as a potential adjunct to peptide-based nerve regeneration

Stem cell-mediated strategies are another emerging approach for peripheral nerve regeneration (103, 104) that may eventually complement peptide-based interventions. Dental-derived stem cells (DSCs) are especially relevant to craniofacial nerve repair owing to their neural crest origin and accessibility (105). Dental pulp stem cells (DPSCs) have garnered the most attention and demonstrate neural-supportive, angiogenic, immunoregulatory, and neurotrophic features (106, 107). Instead of acting only via direct cellular replacement, DPSCs may facilitate regeneration through paracrine signaling, such as the release of growth and neurotrophic factors that affect the local inflammatory niche, vascularization, axonal extension, neuronal survival, and SC functionality (108, 109). Experimental research utilizing non-IAN peripheral nerve models has shown improved myelination, axonal regeneration, and functional recovery after DPSC-mediated therapies (110). Additionally, extracellular vesicles derived from DPSCs (DPSC-EVs) and culture media have exhibited neuroregenerative function (109, 111), indicating that at least part of the treatment impact may be regulated via their secretome instead of long-term cellular engraftment.

Mechanistically, DSCs and regenerative peptides may affect numerous overlapping but potentially complementary elements of the regenerative niche. For instance, DPSC-based paracrine and neurotrophic support could theoretically complement peptide-related cellular viability, inflammatory regulation, microvascular reestablishment, and ECM remodeling. This idea may be especially relevant when cells or their secretome are integrated with nerve guidance conduits or biodegradable matrices, offering a localized regenerative niche alongside controlled delivery of bioactive molecules. Experimental peripheral nerve investigations have already assessed DPSCs in conjunction with nerve conduits, and recent literature also reports DPSC-EVs and conditioned media as cell-free approaches for improving SC activity and peripheral nerve regeneration.

## 8 Clinical evidence and translational gap

Despite a compelling and growing mechanistic rationale, the clinical translation of peptide-based regenerative therapies into dentistry, and specifically into the management of IAN injury, remains in its initial phases. The present evidence base is characterized by a pronounced imbalance between strong preclinical data and limited, heterogeneous human studies. This gap indicates not only the novelty of these treatments but also the intrinsic challenges of translating regenerative approaches across biological systems, injury models, and clinical contexts. A critical assessment of the available evidence shows that while peptide-based therapies exhibit promising biological functionality, the clinical applicability to IAN injury is confined by limited generalizability, lack of validated delivery approaches, uncertain target-site pharmacokinetics, methodological heterogeneity, and model relevance.

### 8.1 Preclinical dominance

Most of the evidence supporting the regenerative activity of peptides, including BPC-157, Tβ4, and ARA-290, is sourced from preclinical investigations (112). These studies have mainly used: (i) rodent models of peripheral nerve injury (sciatic nerve transection or crush injury); (ii) models of metabolic neuropathy, especially diabetic neuropathy; and (iii) non-craniofacial nerve systems, such as peripheral limb and spinal cord nerves (113). These models have offered valuable insights into mechanisms of action, showing improved axonal regeneration, increased myelination, enhanced vascularization, and regulation of inflammatory pathways. Nevertheless, their translational relevance to IAN injury is intrinsically limited (112).

First, the nature of injury varies significantly. Experimental models frequently involve controlled, uniform injuries, including standard crush lesions or clean transections (114). On the contrary, iatrogenic IAN injuries are highly variable and may involve mixed processes, such as chemical irritation, thermal insult, partial transection, and compression (115). This variability complicates direct extrapolation of preclinical outcomes. Second, the anatomical niche of the mandibular canal introduces distinct challenges not replicated in most animal models. The IAN is encased within rigid cortical bone, restricting the capacity for tissue expansion and increasing risk of compressive ischemia (38). Moreover, the proximity to dental structures and surgical sites introduces variables, including the local biomechanical stress, graft materials, and infection predisposition that are rarely considered in experimental models. Third, species-specific variations in nerve regeneration must be accounted for (56). Rodents demonstrate more robust and rapid peripheral nerve regeneration in comparison with humans, potentially overestimating the effectiveness of therapeutic modalities (58). Variations in ECM composition, SC behavior, and immune response further complicate translation (116).

### 8.2 Early human signals

Clinical evidence for peptide-based treatments remains restricted but is progressively emerging, especially for ARA-290 (cibinetide) (113). Early-stage human trials have shown promising outcomes in conditions characterized by small fiber neuropathy, such as sarcoidosis and neuropathy-related nerve damage (88, 113). The revealed outcomes include increased intraepidermal nerve fiber density, enhancement in cutaneous and corneal nerve morphology, and a decrease in neuropathic pain scores (113). These findings indicate that ARA-290 may exert both functional and structural advantages in human peripheral nerve injuries. Crucially, these reports offer preliminary validation that the regulation of the innate repair receptor can translate into measurable clinical effects. Nonetheless, numerous limitations restrict their utilization in IAN injury. First, most of the reports focus on metabolic or systemic neuropathies rather than localized, acute, iatrogenic injuries. The underlying pathophysiology varies substantially, with chronic metabolic dysregulation playing a key role in diabetic neuropathy, while IAN injury is usually an acute traumatic event. Second, the nerves assessed in the reports (distal cutaneous fibers, corneal nerves) vary functionally and structurally from the IAN, which is a mixed sensory nerve situated within a confined osseous canal. Moreover, several clinical reports involve chronic neuropathy, where the therapeutic aim is the re-establishment of long-standing deficits. On the contrary, IAN injury management frequently focuses on acute intervention to prevent advancement to chronic dysregulation. Finally, delivery routes, optimal treatment duration, dosing approaches, and local pharmacokinetic profiles for IAN injuries remain uncharacterized. Especially, it is not known whether systemic delivery can attain therapeutically significant exposure in the bone-enclosed IAN or whether local extended-release approaches will be necessary. Hence, although early human data support the safety and biological plausibility of peptide-based treatments, they fail to establish efficacy in the specific context of dental nerve injury (30, 56, 76).

## 9 A hypothesis-generating, phase-mediated therapeutic model

Considering the multifactorial and temporally evolving biology of IAN injury, a phase-mediated platform may be useful for organizing potential therapeutic targets according to the primary biological pathways transpiring after injury. This model is aimed to be hypothesis-generating instead of prescriptive and should not be taken as an evidence-based intervention method or as support for amalgamating regenerative peptides with traditional agents. No experimental reports have yet defined the efficacy, pharmacological compatibility, optimal sequencing, or safety of these combinations, particularly in IAN injury. IAN regeneration transpires as an active process comprising overlapping phases of degeneration, inflammation, cellular organization, and functional reintegration (117). Hence, the phases outlined below present potential biological windows for prospective research rather than a recommended intervention sequence (Figure 4).

**Figure 4:**
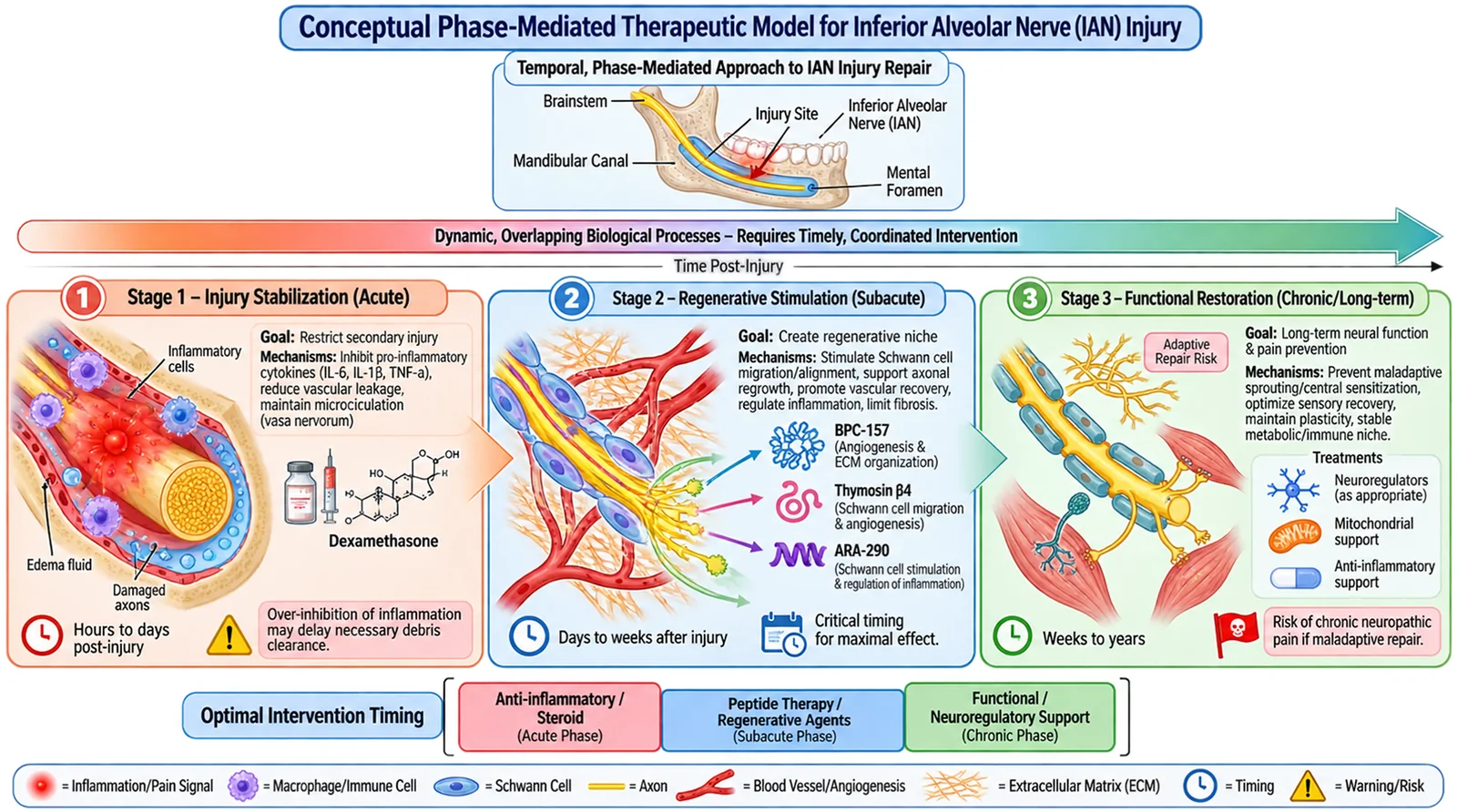
Conceptual phase-mediated therapeutic model for IAN injury. Management is aligned with the biological phases of repair, such as acute injury stabilization, subacute regenerative stimulation, and long-term functional restoration, focusing on timely treatment to optimize regeneration and minimize maladaptive repair.

### 9.1 Stage 1: injury stabilization—restricting secondary injury

In the acute phase, usually covering the first hours to days after injury, the primary aim is to confine secondary neural damage while maintaining conditions needed for resultant repair. Hence, management should focus on controlling excessive inflammation and edema, maintenance of microvascular perfusion, and aversion of extended immunosuppression that could hinder debris clearance. Within this system, time-limited anti-inflammatory therapy may act as an early stabilizing approach instead of a regenerative treatment itself (Figure 4) (38, 51, 57, 58).

### 9.2 Stage 2: regenerative stimulation—re-establishing the microenvironment

As acute inflammation subsides, the therapeutic focus transitions to the reinstatement of a conducive regenerative niche. The principal biological aims comprise SC migration, axonal elongation, revascularization, and regulated ECM remodeling (117). During this phase, the abovementioned complementary impacts of BPC-157, Tβ4, and ARA-290 offer the rationale for their promising utilization as regulators of the regenerative microenvironment rather than as direct “growth stimulants” (68, 72). The timing of these therapies may be pivotal; i.e., therapy before proper regulation of ischemia and inflammation could be ineffective, while excessive delay may allow fibrosis and irreversible degeneration to progress (Figure 4) (58, 117).

Crucially, assigning these agents to a shared regenerative stage does not mean that they must be delivered sequentially or simultaneously. The efficacy and safety of combining BPC-157, Tβ4/TB-500, or ARA-290 with traditional interventions have not been established. Specifically, overlapping activation of angiogenic signaling by BPC-157 and Tβ4/TB-500 needs experimental assessment to identify whether combined exposure yields controlled revascularization or potentially dysregulated/excessive neovascular responses.

### 9.3 Stage 3: functional restoration—integration and long-term results

This stage of nerve recovery comprises functional reintegration and stabilization of regenerated axons, such as reinnervation of target tissues and stabilization of sensory pathways. This phase extends over weeks to years and is marked by consistent remodeling, synaptic refinement, and adaptation with both the central and peripheral nervous systems (117). Central aims include the prevention of chronic neuropathic pain, optimization of sensory repair and discrimination, and support of prolonged neural stability and plasticity. A primary hurdle during this stage is the predisposition to maladaptive repair, such as aberrant axonal sprouting, misdirected reinnervation, and central sensitization, resulting in chronic pain syndromes (58). Treatments to regulate nociceptive signaling, including extended neural inhibition or neuroregulatory approaches, may facilitate alleviating the risk of central sensitization (118). Simultaneously, maintaining a balanced metabolic and inflammatory niche remains vital for ongoing tissue remodeling (119). While the contribution of peptides in this stage is more experimental, their continued impacts on vascular stability, mitochondrial functionality, and low-grade inflammation may help prolong tissue health and functional maturation (Figure 4) (116).

The temporal dimension of this model also intersects with drug delivery. A local extended-release model could theoretically be devised to keep exposure during a defined regenerative window, while systemic delivery would offer less spatial control and may mandate repeated dosing. Nonetheless, since neither the therapeutic concentration nor the optimal duration of exposure has been defined for these peptides in IAN injury, matching a specific delivery platform to a particular regenerative stage remains a research hypothesis instead of a clinical recommendation.

## 10 Limitations of the existing evidence base

### 10.1 Methodological heterogeneity

Beyond the translational constraints, many methodological and regulatory concerns limit the interpretation of the existing evidence base. Across studies, peptide formulations, purity, dosing schedules, administration routes, and treatment duration differ substantially, restricting cross-study comparison and reproducibility.

### 10.2 Uncharacterized peptide-peptide and peptide-drug interactions

Another limitation is the dearth of interaction investigations. While the overlapping biological functions of regenerative peptides offer a conceptual rationale for combinatorial strategies, their simultaneous administration has not been assessed. Potential peptide-peptide and peptide-drug interactions may modify tissue remodeling, vascular activity, immune responses, receptor signaling, and pharmacokinetics in ways that cannot be inferred from reports of individual agents. This concern is especially relevant to BPC-157 and Tβ4/TB-500 since both have been linked with pro-angiogenic signaling. Whether concurrent activation yields beneficial microvascular reestablishment, no supplementary impact, or dysregulated/excessive neovascularization remains un-investigated. Therefore, mechanistic complementarity should not be considered as evidence of clinical synergy or pharmacological compatibility.

### 10.3 Pharmacokinetic and IAN-targeted delivery

Another translational limitation is the absence of IAN-targeted pharmacokinetic and drug delivery data. The local distribution, residency, degradation, and clearance of BPC-157, Tβ4/TB-500, and ARA-290 within the mandibular canal have not been defined. Consequently, discussion of injectable hydrogels, controlled-release biomaterials, or peptide-loaded nerve conduits should be considered future delivery strategies based on broader regenerative-medicine principles rather than validated strategies for IAN injury. Moreover, the limited space of the mandibular canal produces safety concerns, such as pressure-associated compression, vascular compromise, and potential iatrogenic injury, that may not be captured by traditional peripheral nerve models.

### 10.4 Regulatory uncertainty and long-term safety

Regulatory uncertainty represents another primary limitation. Most of these peptides exist in a gray area between clinically approved therapeutics and investigational compounds. While ARA-290 has undergone formal clinical assessment, other agents lack regulatory approval for human application in several jurisdictions. This creates difficulties not only for clinical application but also for the design and funding of large-scale studies. Ethical considerations concerning investigational or off-label application further complicate adoption. Additionally, there is a paucity of long-term safety data, especially regarding the repeated administration, systemic exposure, and potential off-target impacts. Given that several regenerative cascades, including angiogenesis and cell proliferation, are tightly governed, there is a theoretical risk of unintended consequences, such as dysfunctional repair or aberrant tissue growth. Longitudinal reports are hence needed to establish both the durability and safety of outcomes.

### 10.5 Lack of standardized outcome measures

Lastly, the available literature is restricted by inconsistent outcome measures and a lack of standardized evaluation methods. Studies mostly rely on subjective reports of sensory enhancement rather than objective, quantifiable measures. This heterogeneity obstructs comparison across studies and undermines the overall evidence base (Figure 5).

**Figure 5:**
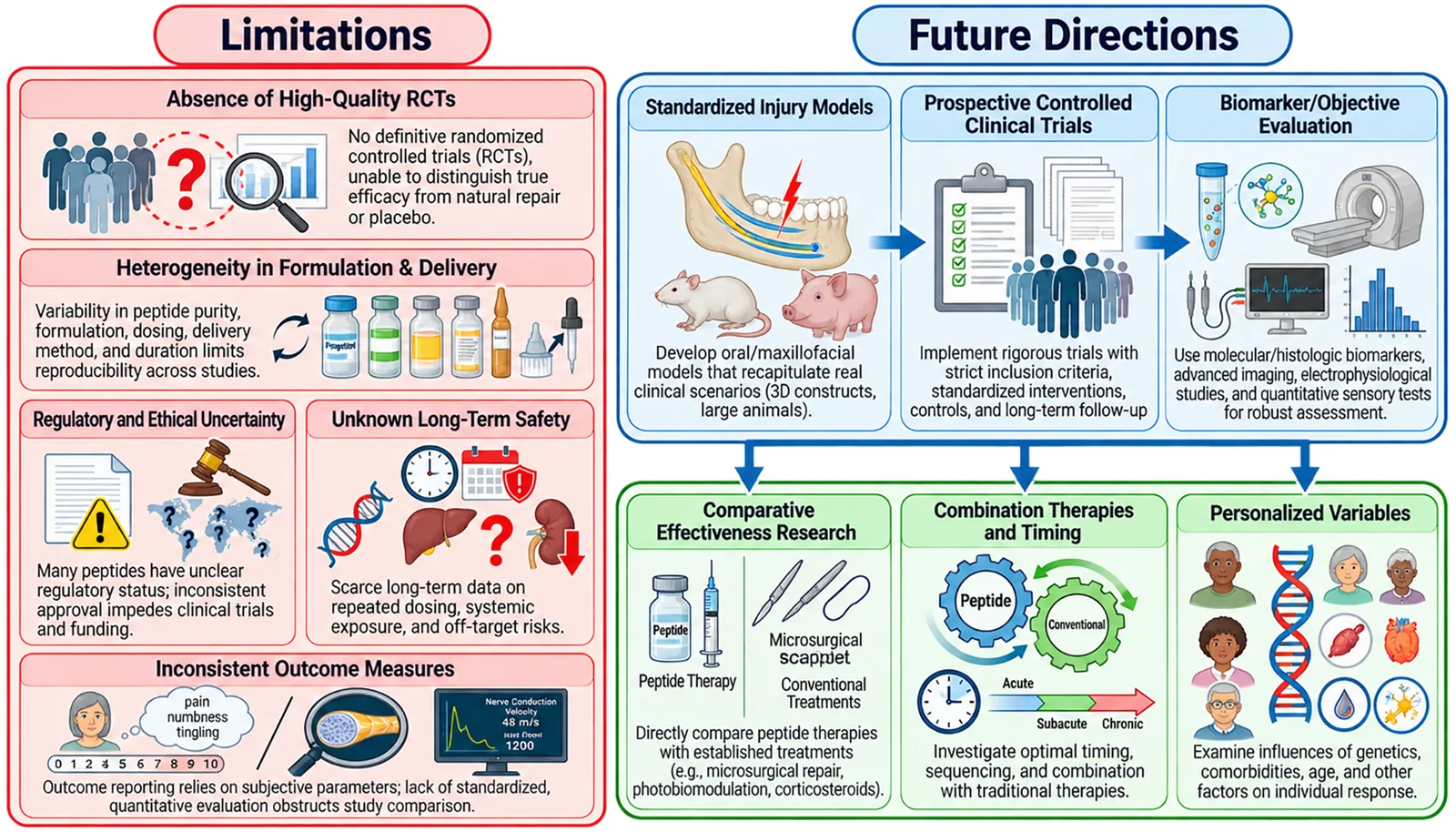
Summary of present limitations and future directions in peptide-mediated interventions for IAN injury.

## 11 Future directions

### 11.1 Clinically relevant preclinical models and controlled clinical trials

Addressing these limitations will mandate a synchronized and methodologically rigorous research plan aiming at connecting the translational gap. Future research should emphasize clinically relevant systems that mimic the biochemical, vascular, and anatomical limitations of IAN injury. Novel 3D bioengineered platforms and large-animal models may offer more representative systems for investigating candidate peptide therapies. Equally pivotal is the implementation of prospective, controlled clinical trials focused particularly on surgically induced and implant-associated IAN injury. Such investigations should integrate: (i) clearly defined inclusion criteria based on injury timing and severity; (ii) standardized therapeutic interventions and dosing regimens; (iii) suitable control or comparator groups; and (iv) longitudinal follow-up to evaluate both short- and long-term outcomes.

### 11.2 Objective biomarkers and outcome assessment

The incorporation of biomarker-mediated evaluation approaches represents another central priority. Objective measures of nerve regeneration, including molecular or histologic markers, high-resolution imaging, electrophysiologic studies, and quantitative sensory testing, will be mandatory for accurately assessing therapeutic efficacy.

### 11.3 Comparative effectiveness and combination approaches

Furthermore, comparative effectiveness investigations are required to position peptide-mediated treatments within the existing therapeutic landscape. Direct comparison with established therapies, including microsurgical repair, photobiomodulation, or corticosteroids, will help identify whether these agents offer synergistic or additive advantage. Such investigations are especially vital for defining their contribution as adjunct versus solo therapies.

### 11.4 Corneal confocal microscopy as an exploratory biomarker of neural regeneration

Corneal confocal microscopy (CCM) may offer a non-invasive method (120) for longitudinal evaluation of small sensory nerve fiber morphology during IAN regeneration studies. CCM allows high-resolution visualization of the corneal sub-basal nerve plexus and quantitative analysis of parameters, including nerve tortuosity, branch density, nerve fiber length, and corneal nerve fiber density (121). These parameters have been utilized to define peripheral small fiber neuropathy, and longitudinal research suggests that CCM can identify nerve regeneration after surgical and pharmacological treatments (122). Notably, CCM does not directly visualize the IAN. Instead, the corneal nerves arise mainly from the ophthalmic division of the trigeminal nerve (123), while the IAN is a branch of the mandibular division (124). Thereby, alterations in corneal nerve morphology should not presently be considered direct evidence of structural IAN regeneration. In future IAN research, CCM could rather be assessed as an exploratory surrogate biomarker of systemic or trigeminal small fiber regenerative activity (125). A prospective report could gather baseline CCM parameters after IAN injury and repeat imaging at pre-specified periods during recovery or peptide-mediated treatment. Alterations in corneal nerve fiber morphology, branching, density, and length could then be associated with IAN-targeted outcomes, such as patient-reported neurosensory recovery, electrophysiological parameters, quantitative sensory assessment of the chin and lower lip, and direct imaging of the injured nerve (126). Demonstrating a reproducible correlation between longitudinal modifications in CCM measures and IAN-targeted functional recovery would be mandatory before CCM could be regarded as a surrogate marker of IAN regeneration. Until such validation, its proposed contribution to IAN injury should be considered hypothesis-generating and complementary instead of as a direct measure of repair.

### 11.5 Injury-severity classification in prospective research

Furthermore, future IAN-targeted research should categorize participants based on injury severity utilizing a standardized system. Pooling neurotmetic, axonotmetic, and neurapraxic injuries within a single treatment population could hinder treatment impacts since these injuries vary considerably in spontaneous regenerative capacity, structural integrity, and necessity for surgical reconstruction.

### 11.6 In-silico modeling for temporal and dose optimization

Considering the dynamic and intertwined nature of peripheral nerve regeneration, in-silico methods may offer a useful model for integrating the pathway-targeted, dose-responsive, and temporal effects of candidate regenerative peptides. Computational network models could incorporate central pathways involved in IAN repair, such as macrophage polarization, SC activity, inflammatory cytokine pathways, oxidative stress, angiogenesis, ECM remodeling, and axonal regeneration, and consequently map how these mechanisms evolve across varying stages of nerve repair. Such models may be especially relevant to BPC-157, Tβ4/TB-500, and ARA-290 since their proposed biological functions intersect at multiple regenerative cascades while varying in their primary immunomodulatory, cytoskeletal, and vascular impacts.

Incorporation of pharmacokinetic/pharmacodynamic (PK/PD) modeling into systems biology or network-mediated strategies could further aid in predicting how treatment propagation, dosing interval, delivery route, local tissue exposure, and peptide concentration impact regenerative pathways within the mandibular canal. Crucially, such frameworks could analyze whether a given therapy has a narrow temporal window in which its biological impacts are favorable, or whether extended or excessive pathway stimulation could theoretically interfere with consequent stages of repair. Moreover, computational simulations could evaluate potential interactions among traditional treatments and regenerative peptides before combinations are assessed experimentally, such as overlapping impacts on angiogenic and inflammatory signaling.

However, in-silico predictions should be interpreted as hypothesis-generating instead of evidence of treatment efficacy. Their reliability will rely on the quality of experimental data utilized for model parameterization and validation, which is presently restricted for IAN-targeted peptide temporal biological responses, dose-dependent associations, and peptide pharmacokinetics. Hence, an iterative approach incorporating computational predictions into IAN-associated *in-vitro*, ex-vivo, and *in-vivo* validation may facilitate prioritizing candidate therapeutic combinations, therapeutic windows, and doses for subsequent experimental analysis while decreasing empiric testing of poorly supported regimens.

### 11.7 Dental stem cells as a potential adjunct to peptide-based nerve regeneration

However, the potential synergy between the regenerative peptides discussed in this study and DSC treatment remains hypothetical. To our knowledge, there is presently inadequate evidence showing that BPC-157, Tβ4/TB-500, or ARA-290 combined with DPSCs or other DSCs yields synergistic or additive regeneration of the IAN. Overlapping impacts on angiogenesis, immune modulation, and tissue remodeling could theoretically be advantageous but could also generate unpredictable or redundant biological responses. Moreover, cell viability, differentiation, interactions, spatial retention, dose, delivery, and immunogenicity between transplanted cells and peptide-based pathways would need systematic research. Hence, DSCs should currently be regarded as a potential complementary regenerative system for prospective research instead of an established synergistic therapy with regenerative peptides. IAN-targeted investigations comparing peptide treatment, DSC intervention, and their amalgamation will be needed to identify whether true biological synergy exists.

### 11.8 IAN-specific drug delivery and pharmacokinetic optimization

Prospective research should regard drug delivery as a key element of therapeutic development for IAN injury. Comparative investigations are required to identify whether biodegradable extended-release constructs, injectable hydrogel-mediated depots, local bolus injection, or systemic delivery offer the most appropriate balance between target-site safety and exposure. Such reports should quantify peptide concentration within the injured nerve and surrounding mandibular tissues over time rather than depending only on plasma pharmacokinetics. Pharmacodynamic-pharmacokinetic analyses should define systemic spillover, degradation kinetics, tissue residency, exposure duration, response interactions, and local concentration. Large animal models that mimic the osseous confinement and dimensions of the human mandibular canal would be especially relevant for this purpose. Moreover, delivery systems should be assessed for the potential to generate additional compression, effects on the inferior alveolar neurovascular bundle, mechanical compatibility with the canal, swelling, degradation, and injectability. For severe injuries needing microsurgical exploration, peptide-containing nerve conduits or biodegradable matrices could be assessed as adjuncts to traditional repair. Contrarily, for injuries managed non-surgically, research should define whether systemic administration can produce sufficient local neural exposure without unwanted off-target impacts. These questions must be addressed before a clinically applicable peptide delivery system for IAN injury can be established (Figure 5).

## 12 Conclusion

Regenerative peptides constitute a biologically plausible; however, investigational approach for IAN injury. Their primary attraction is the potential to regulate intertwined elements of the regenerative niche instead of a single downstream pathway. Nonetheless, the existing evidence is sourced mainly from preclinical systems and clinical outcomes measured outside the specific setting of IAN injury, preventing conclusions about treatment efficacy. Successful translation will mandate IAN-relevant experimental systems, standardized preparations and outcome measures, pharmacokinetic characterization, and rigorously controlled clinical studies. A phase-aligned method connecting treatment pathways with the evolving biology of IAN injury may offer a useful system for these studies and facilitate defining whether peptide-mediated interventions eventually have a role alongside established management approaches.
